# Luminance, Colour, Viewpoint and Border Enhanced Disparity Energy Model

**DOI:** 10.1371/journal.pone.0129908

**Published:** 2015-06-24

**Authors:** Jaime A. Martins, João M. F. Rodrigues, Hans du Buf

**Affiliations:** 1 Vision Laboratory (FCT), ISR-LARSyS, University of the Algarve, Faro, Portugal; 2 Vision Laboratory (ISE), ISR-LARSyS, University of the Algarve, Faro, Portugal; The Ohio State University, Center for Cognitive and Brain Sciences, Center for Cognitive and Behavioral Brain Imaging, UNITED STATES

## Abstract

The visual cortex is able to extract disparity information through the use of binocular cells. This process is reflected by the Disparity Energy Model, which describes the role and functioning of simple and complex binocular neuron populations, and how they are able to extract disparity. This model uses explicit cell parameters to mathematically determine preferred cell disparities, like spatial frequencies, orientations, binocular phases and receptive field positions. However, the brain cannot access such explicit cell parameters; it must rely on cell responses. In this article, we implemented a trained binocular neuronal population, which encodes disparity information implicitly. This allows the population to learn how to decode disparities, in a similar way to how our visual system could have developed this ability during evolution. At the same time, responses of monocular simple and complex cells can also encode line and edge information, which is useful for refining disparities at object borders. The brain should then be able, starting from a low-level disparity draft, to integrate all information, including colour and viewpoint perspective, in order to propagate better estimates to higher cortical areas.

## 1. Introduction

Disparity plays an important role in our perception of the environment, giving us precious information for survival. Our brain extracts it from the information that reaches the hypercolumns of V1 via the Lateral Geniculate Nucleus (LGN), which relays information of the left and right retinae. At this early stage, disparity is already key for broad and precise motor control (e.g., walking/running while avoiding obstacles, eye-hand coordination while picking up a pencil), low- and high-level Focus-of-Attention (FoA), object and background segregation, as well as recognition, even with partial occlusions [1].

Computer vision research has significantly advanced the state-of-the-art in disparity estimation models, with many different approaches and applications [2]. However, there is a significant lack of biologically motivated models that computationally implement the Disparity Energy Model (DEM), which integrates key biological evidence from research on the cat’s visual cortex and pathways by [3], and more recently from the rhesus monkey’s visual cortex [4]. Alternative models also exist for building and combining disparity energy neurons [5]. The DEM allowed to explain how neurons tuned to horizontal disparities can have the implicit ability to discriminate vertical disparities [6]. This ability is an emerging property from a neuronal system tuned to horizontal disparities, by decoding vertical ones as a deviation from the expected neuronal responses. This ability also illustrates how the neuronal system can encode much richer information than would be expected and, at the same time, concentrate neuronal resources on the most common cases while keeping the possibility of encoding rare ones.

Most DEM computational implementations found in the literature were unable to give good results on real-world images. Therefore, we first focused on building upon a state-of-the-art theoretical DEM implementation by [6] until we could reliably extract disparity estimations from real-world data. This was documented in [7]. It is still the only DEM-based method ranked on the Middlebury Stereo Evaluation Website [8], against 153 other disparity methods.

Some authors have proposed alternative biological models which are not based on the DEM, e. g., [9] combining geometric information and local edge features, [10] using multiscale lines and edges to retrieve a disparity wireframe model of the scene—the Line and Edge Disparity Model (LEDM) which is further explored in this paper in §5.1—and also du Buf et al. [11], employing the phase differences of simple cell responses to the left and right views. The latter model is often applied to real-world problems, although it has been shown to be very imprecise in terms of localisation of depth transitions.

Most DEM research has considered theoretical or synthetic data, while biological models applied to real-world scenes appeared only recently [7, 9, 10, 12]. This is mainly due to the fact that computational DEM implementations are usually focused on evaluating theoretical results using very specific stimuli, like bar/grating patterns or random-dot stereograms [6], or in psychophysical experiments [4].

In this paper, we propose a disparity map composed of different cell maps built on top of each other, each refining the previously extracted disparity. We also propose that the first, rough disparity (disparity *gist*) is provided by the DEM model [7], after which refinements based on colour, perspective correction (viewpoint) and border information are integrated to achieve the final disparity map. Although the model is still feed-forward or bottom-up, in the future it can be supplemented by feedback loops from higher visual areas V2 and V4 in order to further improve results [1].

In our improved DEM implementation we use two neuronal populations for obtaining disparities:

1. An *encoding population* which uses a set of binocular neurons with a diverse range of cell parameters, e. g., horizontal disparities, spatial frequencies and orientations. This population is trained on random-dot stereograms in order to learn activity codes for many different disparities. The method is similar to that of [6], which is based on the DEM model of [3], with proper normalisation to yield local correlations with neighbourhood weighting [13–15]. Finally, the population is applied to real stereograms in order to obtain local activity codes. This is further explained in §3.1.
2. A higher-level *decoding population* which compares a local activity code, at each image position, with all learned (trained) activity codes, for estimating local disparity. This is further explained in §3.2. Basically, this second population implements a template-matching process similar to those of [16] and Read [6]. This initial DEM model (disparity gist) is then integrated with colour and different viewpoints (§4), and finally with object border information retrieved from the multi-scale line and edge disparity model (LEDM) [10] and low-level processes from object salience research [17] (§5).

Our main contributions in this paper are: (a) Improving previous DEM results in real-world images. (b) The integration of the DEM model with luminance, colour information and viewpoint perspective correction. (c) The integration of two disparity models DEM and LEDM, to improve object boundary precision of the DEM. (d) The integration of different layers of disparity cell maps, with each layer improving the results from layer to layer. (e) The quantitative evaluation of results with real-world scenes, showing that the model can compete with state-of-the-art computer vision algorithms.

## 2. Disparity-sensitive cells

The primary visual cortex (V1) is composed mostly of simple, complex and end-stopped (hypercomplex) cells arranged into ocular dominance hypercolumns. Computationally, the receptive fields (RFs) of monocular simple cells can be modelled by Gabor wavelets [7, 18, 19], with parameters to specify orientation *θ*, spatial frequency *f* (or the wavelength *λ* = 1/*f*), receptive field size *σ* and spatial phase *ϕ*, which will be discussed below. We can then model *binocular* simple cells using pairs of *monocular* simple cells with either a position- or phase-shift between RFs (or a combination of both), signalling disparity when both RFs of the binocular cell are fully excited. However, binocular simple cells are also sensitive to stimulus contrast and pattern position within their RFs [3, 18], which makes them unsuitable as disparity detectors.

In contrast, binocular *complex* cells can solve these problems, as there are no separate excitatory and inhibitory subregions within their RFs, making them only sensitive to position, orientation and stimulus size [20]. They also show other desirable properties like sensitivity to fine disparities, immunity to anti-correlated stimuli [3] and they respond accurately to dynamic random-dot stereograms [21]. Two binocular simple cells *S*_1_ and *S*_2_ can be combined into a phase-independent binocular complex cell, provided that their phase difference ∣*ϕ*_*S*_1__ − *ϕ*_*S*_2__∣ equals *π*/2. Therefore, the response of a binocular complex cell can be obtained by summing the responses of two binocular simple cells with phases in quadrature.

Mathematically, two monocular RFs can be used to model a binocular *simple* cell, with the same size, orientation and spatial frequency, but with different phases *ϕ* and/or RF positions on the retina (Δ*x*, Δ*y*) [22]. The left (*ρ*_*L*_) and right (*ρ*_*R*_) RFs of binocular simple cells are then defined by

$$\begin{array}{c} \rho_{L,R}\left(x,y;\theta ,\sigma ,f,\phi ,\Delta \phi \right)=\exp(-\frac{{\dot{x}}_{L,R}^{2}+{\dot{y}}_{L,R}^{2}}{2\sigma^{2}})\cos(2\pi f{\dot{x}}_{L,R}+\phi \pm \frac{\Delta \phi }{2}). \end{array}$$ (1)

Since we will use phases in quadrature *ϕ* ∈ {0, −*π*/2} and both *ρ*_*L*_ and *ρ*_*R*_ actually consist of two RFs: the sine and cosine components. In Eq. 1, $\dot{x}$ and $\dot{y}$ are the coordinates relative to the binocular cell’s centre, which is (0, 0) at the fovea, and rotated according the cell’s preferred orientation *θ*:

$$\begin{array}{c} {\dot{x}}_{L,R}=x_{L,R}\cos\theta +y_{L,R}\sin\theta \end{array}$$ (2a)

$$\begin{array}{c} {\dot{y}}_{L,R}=-x_{L,R}\sin\theta +y_{L,R}\cos\theta . \end{array}$$ (2b)

The left disparity viewpoint is used as reference, requiring the use of binocular cells with left predominance. The main reason for using the left view is that it is often used for defining the ground-truth of real scenes, thus allowing for a quantitative analysis of experimental results. Mathematically, the offset coordinates Δ*x* and Δ*y*, which correspond to the cell’s preferred horizontal and vertical disparities, are defined as follows: when the activity code is trained (learned) with random-dot stereograms, the left RF is centred at (0, Δ*y*) and the right one at (−Δ*x*, Δ*y*). When the cells are applied at all input stereogram positions, then (*x*_*L*_, *y*_*L*_) = (*x*, *y* + Δ*y*) and (*x*_*R*_, *y*_*R*_) = (*x* − Δ*x*, *y* + Δ*y*). We note that Δ*y* = 0 is taken for all cells, as vertical disparity in the fovea is zero [22]. For a detailed mathematical transformation from monocular to binocular simple cells see [18].

## 3. Luminance Disparity-Energy Model

In this section, we describe the lL-DEM or L-DEM, and show how disparity maps can be extracted by exploiting binocular cell responses and comparing them with previously learned stimuli, using cells sensitive only to luminance variations. The L-DEM was first presented in Martins et al. [7] and is adapted partly for this section, serving here to provide a performance baseline. Understanding this model is also fundamental for understanding all further improvements described in this paper.

For the L-DEM implementation, we use two neuronal populations: (1) an encoding population and (2) a higher-level decoding population. As explained above, for presenting our stereo results we use by default the reference viewpoint (image) of the left eye.

### 3.1 Disparity encoding population

For the encoding population’s binocular simple cells defined in Eq. (1), we selected RF parameters based on [6]:

(a) Orientations *θ*_*i*_ ∈ (*i* × *π*)/*N*_*θ*_, with the number of orientations *N*_*θ*_ = 8. Our empirical tests showed that using more orientations yielded slightly better disparity estimates, but increases the total cell population. Using eight orientations is a good compromise.
(b) Receptive field sizes (scales) $\sigma \in \left\{2\sqrt{2},\;2,\;\sqrt{2}\right\}$. These are scaled by a factor of $\sqrt{2}$, as is the spatial frequency. Empirical results showed that bigger sizes increase the blur at objects’ border regions and smaller sizes lead to errors in disparity estimates.
(c) Spatial frequencies $f\in \left\{\sqrt{2}/8,\;1/4,\;\sqrt{2}/4\right\}$ cycles per pixel. These values are proportional to RF size by *ωσ* = *π* or *f* = 1/2*σ*. The frequency bandwidth for the three scales was 1.14 octaves.
(d) RF phases *ϕ* ∈ {0, −*π*/2}, since only two values are needed to build a phase-invariant binocular complex cell from two binocular simple cells [3].
(e) RF horizontal position disparity Δ*x* ∈ {0, …, 59} in steps of 1 pixel.
(f) RF phase disparity Δ*ϕ* = 0, implying no extra phase difference between the left and right RFs of each simple cell (equal phases *ϕ* for both). It is to be expected that in naturally occurring images, the maximum response of a phase-shift disparity neuron is elicited when there is a different pattern of the same stimulus in the left and right RFs, something that never occurs in the real world [4, 5]. Our empirical tests also showed that the use of phase differences—odd-symmetric disparity tuning curves—did not add significant information and sometimes even degraded the quality of disparity estimates. Other alternative roles for neurons tuned to phase disparities are explained further in [23].

In total, the above selection yields a population of 8_*θ*_ × 3_*σ*, *f*_ × 2_*ϕ*_ × 60_Δ*x*_ × 1_Δ*ϕ*_ = 2880 binocular simple cells as inputs for 1440 binocular complex cells; see below. The values were chosen to replicate physiological parameters of real cells, for yielding precise disparity estimates in real-world images. The disparity encoding population is then built and trained as follows, based on Read [6]:

#### Stereo energy coding

Responses of the left and right RFs of binocular simple cells (*v*_*L*_ and *v*_*R*_) are obtained by convolving (*) the RFs with the corresponding left and right grayscale images *I*_*L*,*R*_(*x*, *y*):

$$\begin{array}{c} v_{L,R}\left(x,y\right)=I_{L,R}\left(x,y\right)*\rho_{L,R}\left(x,y\right). \end{array}$$ (3)

To simplify notation, below we skip (*x*, *y*). *I*_*L*,*R*_ are obtained from sampling an RGB colour stereogram using physiologically perceived weights from the luminance Y channel of the CIE XYZ colour-space, which closely resembles human colour perception: *I*_*L*,*R*_ = 0.2989 · *R*_*L*,*R*_ + 0.5870 · *G*_*L*,*R*_ + 0.1140 · *B*_*L*,*R*_.

At each image position, the response *S* of a binocular simple cell combines the squared responses of the left and right RF components [3, 18]:

$$\begin{array}{c} S={\left(v_{L}+v_{R}\right)}^{2}=v_{L}^{2}+v_{R}^{2}+2\;v_{L}v_{R}. \end{array}$$ (4)

*S* can be split into the monocular term $M=v_{L}^{2}+v_{R}^{2}$ and the binocular term *B* = 2 *v*_*L*_ *v*_*R*_. Biologically, this can be realised by combining the outputs of two energy neurons with phase disparities *π* apart. If such neurons are identical except for their phase disparities, then the first one computes (*M* + *B*) and the second (*M* − *B*). Both *M* and *B* are then available from the sum and difference of the two responses, i. e., 2*M* and 2*B* [6].

For obtaining the local stereo energy *E* of a binocular complex cell which is invariant to the phases of local patterns in the input, one can either sum the responses of (a) many binocular simple cells with scattered phases *ϕ* in [0, 2*π*], or (b) only two cells with phases in quadrature. We could therefore apply the second case with *ϕ* ∈ {0, −*π*/2}: *E* = Σ_*ϕ* = {0, −*π*/2}_ *S*_*ϕ*_. This stereo energy *E*, for each frequency, orientation and disparity, can be related to the cross-correlation between filtered and windowed images [15]. However, the local stereo energy *E* cannot be used directly to estimate disparities, as it also reflects monocular energy (stimulus contrast inside each RF) along with binocular energy (stimulus disparity between RFs). This shortcoming is addressed below by using *spatial pooling* and *effective binocular correlation*.

#### Spatial pooling

Complex cells are normally modelled by taking the square root of the sum of the squared responses of the sine and cosine components of the simple cells. This implies that the RF size of such complex cells is equal to that of the simple cells: the same Gaussian. However, RFs of real binocular complex cells are larger than those of simple cells [18]. Therefore we apply this property by averaging *M* and *B*, using grouping cells with a Gaussian RF: *G* ^sp^(*x*, *y*) = *k* exp (−(*x* ^2^ + *y* ^2^)/2*σ* ^2^). The normalisation factor *k* = 1/(2*πσ* ^2^) and *σ* equals the RF size of the corresponding simple cells: $\sigma \in \left\{2\sqrt{2},\;2,\;\sqrt{2}\right\}$. This yields, for the two phases, $M_{\phi }^{\text{sp}}=G^{\text{sp}}*M_{\phi }$ and $B_{\phi }^{\text{sp}}=G^{\text{sp}}*B_{\phi }$. This pooling operation involves using simple grouping cells with a dendritic field size defined by *σ* and it is crucial to stabilise results in case of real-world images with noise and non-uniform disparity ranges.

#### Effective binocular correlation

In order to differentiate monocular energy from binocular energy, it is necessary to use normalised binocular correlation detectors [6, 13–15]. These detectors respond maximally (+1) when the left and right RF views are identical, and minimally (−1) when one RF view is an inverted-contrast version of the other. They are implemented by dividing the pooled binocular term by the pooled monocular term, after which the result is pooled once more for increasing robustness:

$$\begin{array}{c} \psi^{\text{sp}}=G^{\text{sp}}*(\frac{\sum_{\phi =\{0,-\pi /2\}}B_{\phi }^{\text{sp}}}{\sum_{\phi =\{0,-\pi /2\}}M_{\phi }^{\text{sp}}}). \end{array}$$ (5)

The value of *ψ* ^sp^ relates to the correlation between local, filtered regions of the left and right views [23]. The population of binocular correlation detectors *ψ* ^sp^ is used for encoding disparity in the model. Disparities estimated by using the effective binocular correlation instead of the local stereo energy *E* are immune to the detrimental effect of monocular contrast, allowing the extraction of disparity from peaks in the population’s activity code. *ψ* ^sp^ has also the useful property that it exactly equals 1 when the actual disparity matches a cell’s preferred disparity [6]. Please recall that *ψ* ^sp^ is the short notation for $\psi_{f,\theta ,\Delta x}^{\text{sp}}(x,y)$, i. e., there are three scales, eight orientations and 60 horizontal position disparities, hence 1440 binocular correlation cells which are later applied at all image positions.

#### Learning the population code

**Fig 1:**
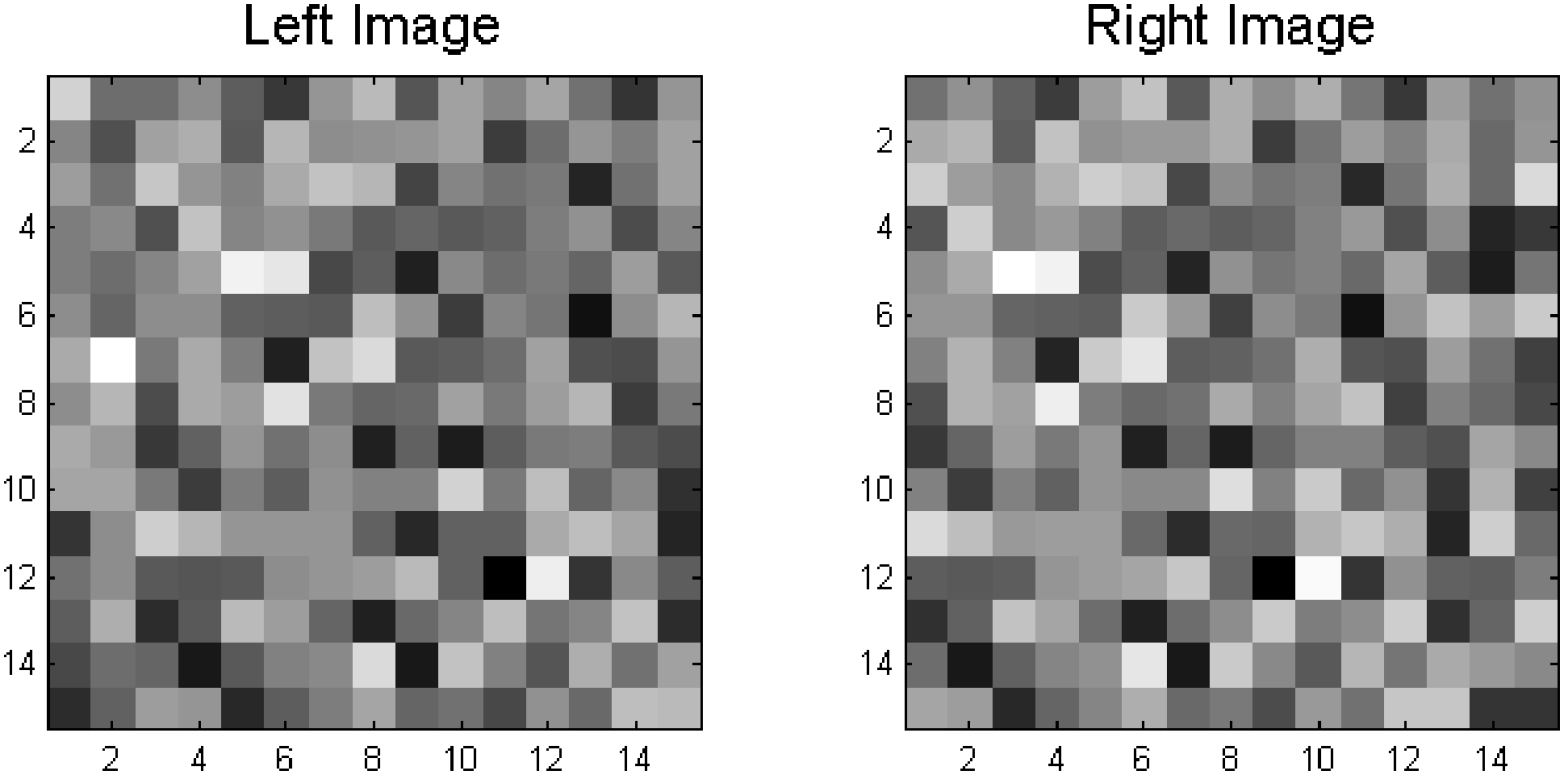
Example of a 15 × 15 random-dot stereogram used in training, with a uniform 2-pixel shift and thus horizontal disparity Δ*x*_stim_ of 2.

We trained the energy model to discriminate horizontal stimulus disparities (Δ*x*_stim_) ranging from 0 to 59 pixels with a stepsize of 1 pixel. Population activity codes were gathered from cell responses to stimuli with known disparities: random-dot stereograms with an uniform disparity, sampled randomly from a Gaussian distribution with zero mean and unit standard deviation, with a Δ*x*_stim_ horizontal offset between the left and right images. Offset gaps were also filled with randomly sampled pixels; see Fig 1. For each Δ*x*_stim_ step we generated 1000 random-dot pairs. Hence, training involved 60,000 stereograms; for details see Martins et al. [7]. For each stereogram, with *I*_*L*,*R*_ the left and right views, we applied Eq (3) and Eq (4), but only at the centre of the left and right images of each stereogram. The values of *ψ* were computed without spatial pooling, i. e.,

$$\begin{array}{c} \psi =\frac{\sum_{\phi =\{0,-\pi /2\}}B_{\phi }}{\sum_{\phi =\{0,-\pi /2\}}M_{\phi }}, \end{array}$$ (6)

because the results are pooled over 1000 random-dot stereograms for each disparity.

During training, and later when applying the population to real images, the effective binocular correlations *ψ* and *ψ* ^sp^ are encoded as a *mean spike count*,

$$\begin{array}{c} \Psi =(1+\psi )\;u, \end{array}$$ (7)

where *u* = 8 is the average number of spikes elicited by a binocularly uncorrelated stimulus within the temporal discrimination window. We used parameters similar as [6], with typical values of *u* around 8 spikes, assuming a firing rate for the optimal disparity of 100 Hz and a temporal window of 160 ms. This yields values of Ψ in the range [0,2*u*], where 2*u* represents the mean number of spikes that neurons tuned to a specific disparity will fire in the presence of a perfect binocular stimulus of that disparity (maximum correlation).

Finally, Ψ was averaged [A(·)] over the 1000 different stereograms for each Δ*x*_stim_, which serves to eliminate random stimulus-dependent noise. This yields an activity code for each trained horizontal disparity Δ*x*_stim_:

$$\begin{array}{c} W_{f,\theta ,\Delta x}^{\;\Delta x_{\text{stim}}}=\text{A}(\Psi_{f,\theta ,\Delta x}^{\;\Delta x_{\text{stim}}}). \end{array}$$ (8)

In summary, *W* represents the number of spikes produced by neurons tuned to frequencies *f*, orientations *θ* and horizontal disparities Δ*x*, averaged over all 1000 stimuli with the same uniform disparity Δ*x*_stim_. The population code thus consists of 1440 binocular correlation cell responses (3 scales, 8 orientations and 60 horizontal position disparities) for *each* of the 60 different horizontal stimulus disparities Δ*x*_stim_ of the random-dot stereograms. The adaptation and learning of the encoding cell population to discriminate disparities can be thought of as kin to visual learning in early childhood, assuming that basic neural circuitry is the result of evolution, or, at least, needs adequate training to reach its full potential.

### 3.2 Disparity decoding population

As mentioned before, learning is done only once and in the centre of the random-dot stereograms. After training, the encoding population can then be applied at all pixel positions (neighbourhoods) of real world input stereograms, excluding the border region. The disparity at each position is estimated by comparing the activity code there with all learned codes. This is done by a second, higher-level *decoding* population. The disparity assigned to each pixel position is the disparity of the best-matching code. Local disparity estimation is a simple matching process [16]: the input code of 1440 responses is matched or correlated with the 60 sets of 1440 trained codes. The final output is selected by the decoding population by a winner-takes-all strategy. Biologically, this probably involves associative memory, which can also be based on a training process [24].

The matching process uses 60 correlation cells (“Corr”) which compare $\Psi_{f,\theta ,\Delta x}^{\text{sp}}(x,y)$ with $W_{f,\theta ,\Delta x}^{\Delta x_{\text{stim}}}$, i. e., the 1440 spike counts at each image position with all previously learned 60 sets of 1440 spike counts:

$$\begin{array}{c} r_{\Delta x_{\text{stim}}}\left(x,y\right)={\left[\text{Corr}(\Psi_{f,\theta ,\Delta x}^{\text{sp}}\left(x,y\right),W_{f,\theta ,\Delta x}^{\;\Delta x_{\text{stim}}})\right]}^{+}, \end{array}$$ (9)

where [·]^+^ is half-wave rectification. This avoids the problem of disparity in anti-correlated stereograms by setting any negative correlations to zero [25]. Note that *r*_Δ*x*_stim__ is a vector of 60 correlation values, each related to a specific Δ*x*_stim_ disparity that the population was trained to recognise, from 0 to 59. The maximum correlation yields the luminance-disparity map $D^{\text{L}}(x,y)=\underset{\Delta x_{\text{stim}}}{\text{arg max}}[r_{\Delta x_{\text{stim}}}(x,y)]$. Biologically, this corresponds to the activation of a single disparity cell at each position, inhibiting the other 59 cells. Mathematically, the implemented matching process (Corr) is the Pearson product-moment correlation coefficient with A[·] the average, *σ*_Ψ_ and *σ*_*W*_ the standard deviations of all 1440 responses:

$$\begin{array}{c} r_{\Delta x_{\text{stim}}}\left(x,y\right)={\left[\frac{\text{A}[\Psi^{\text{sp}}\left(x,y\right)W]-\text{A}[\Psi^{\text{sp}}\left(x,y\right)]\text{A}(W)}{\sigma_{\Psi^{\text{sp}}\left(x,y\right)}\sigma_{W}}\right]}^{+}. \end{array}$$ (10)

### 3.3 Experimental results

The obtained results for this method were first published in Martins et al. [7], where we tested the Luminance Disparity Energy Model (L-DEM) on various reference stereograms from the Middlebury stereo evaluation set. These are: *tsukuba*, *venus*, *teddy* and *cones* [26, 27], *aloe* and *cloth3* of the 2006 dataset, and *dolls*, *moebius* and *reindeer* of the 2005 dataset [28].

**Fig 2:**
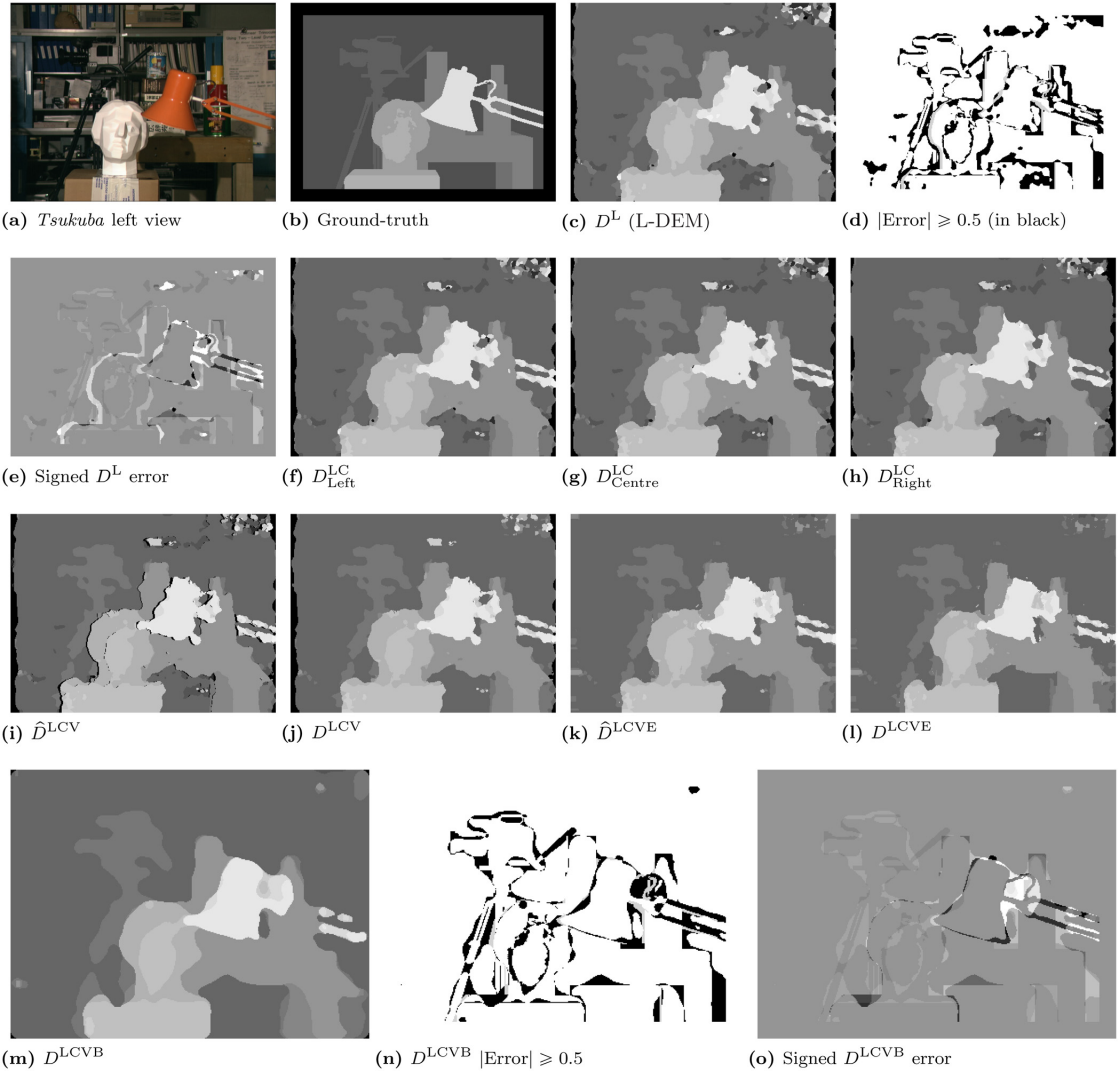
Disparity results in the cell map layers for the *tsukuba* [27] stereo pair. **(a)**: *Tsukuba*’s left view. **(b)**: Ground-truth. **(c)**: *D* ^L^ (L-DEM) result. **(d)**: Bad pixels (black) with an absolute disparity error ≥ 0.5 and **(e)**: signed disparity error returned by the Middlebury evaluation test [8]. **(f)**: Left–viewpoint $D_{\text{Left}}^{\text{LC}}$. **(g)**: Centre–viewpoint $D_{\text{Centre}}^{\text{LC}}$. **(h)**: Right–viewpoint $D_{\text{Right}}^{\text{LC}}$. **(i)**: Left-viewpoint corrected ${\hat{D}}^{\text{LCV}}$. **(j)**: Background and occlusion corrected *D* ^LCV^. **(k)**: Line and edge region enhanced ${\hat{D}}^{\text{LCVE}}$. **(l)**: Object border enhanced *D* ^LCVE^. **(m)**: The final disparity map *D* ^LCVB^, after median smoothing. **(n)**: Bad pixels (black) with absolute disparity error ≥ 0.5. **(o)**: Signed disparity error of *D* ^LCVB^. Images (a) and (b) are reprinted from [26] under a CC BY license, with permission from Daniel Scharstein, original copyright 2002.

For reference, Fig 2(a)-2(e) shows the L-DEM results for the *tsukuba* stereo pair [7, 27]. This algorithm was able to obtain good results for the Middlebury evaluation test (ranked there as “BioDEM”) [7], which are detailed in §6. We will compare further disparity improvements using these results as baseline.

## 4. Luminance, Colour and Viewpoint DEM

This section addresses an improved disparity model, the Luminance, Colour and Viewpoint Disparity Energy Model(LCV-DEM), which integrates colour and viewpoint (perspective) information to increase accuracy of the L-DEM.

Research involving the chromatic representation in area V1 has shown that cone responses from the retina turn into three relatively independent spatio-chromatic colour channels after the LGN [29], which are then transformed in several neural pathways, mixing colour responses with those of other cells [30]. The majority of neurons in V1 seem to respond to pure isoluminant stimuli (i. e., they are colour sensitive even in the absence of luminance changes), and around 50% of all neurons are sensitive to both luminance and isoluminant stimuli. They are classified as either “colour-luminance” or “luminance-preferring” cells with a varying degree of cone opponency [31]. There is also evidence that chromatic features are useful for binocular correspondence in complex images, suggesting the possibility of independent contributions from both luminance and colour channels [32, 33]. In addition, it has been reported that there exist V2 neurons of macaques that are sensitive to both colour and disparity, supporting the notion that the primate visual system combines disparity and colour as early as in area V2 [34].

For the LCV-DEM implementation we initially chose the LMS colour space, which mimics the trichromatic neuronal encoding of cone responses after the LGN [30]. However, the results obtained with the LMS colour space were not significantly better than those with a simple variation of RGB (each channel codes both luminance and colour). This is not surprising. Since neuronal cells have so many different combinations of luminance or colour predominance, the system is able to be independent of the colour method used, as long as there is enough variety of weight predominance between the different colour channels. We did, however, get better results when using physiologically perceived colour weights for encoding luminance (the Y channel of the XYZ colour space), suggesting that not only disparity is heavily luminance based, but also that it depends on luminance being perceptually representative of the scene being observed.

### 4.1 Disparity encoding population

The extended model uses the same population parameters as L-DEM, defined in §3.1, with, in addition to points (a) to (f), point

(g) RF dominance *μ* ∈ {Left, Centre, Right}: three values of binocular RF dominance, representing three possible configurations for RF organisation around a centre point, as further explained below.

We can improve disparity estimates by using two more RF dominances. As previously mentioned, the binocular simple cell RFs are defined by *ρ*_*L*,*R*_ in Eq (1), where $\left(\dot{x},\dot{y}\right)$ are offset coordinates relative to the centre (0,0) and rotated to the cell’s preferred orientation according to Eq (2). For *μ* = Left we use (*x*_*L*,*R*_, *y*_*L*,*R*_) as shown in §2, representing both RFs centered around −Δ*x*/2. For *μ* = Centre the RFs are equidistant from (0,0) and their coordinates are (*x*_*L*_, *y*_*L*_) = (*x* + Δ*x* /2, *y*+Δ*y*/2) and (*x*_*R*_, *y*_*R*_) = (*x*−Δ*x*/2, *y* − Δ*y*/2). For *μ* = Right the RFs are shifted to the right and centered at Δ*x*/2, resulting in coordinates (*x*_*L*_, *y*_*L*_) = (*x*+Δ*x*, *y*+Δ*y*) and (*x*_*R*_, *y*_*R*_) = (*x*, *y*).

#### Stereo energy coding

The LCV-DEM model also employs pairs of binocular simple cells in quadrature in order to construct phase-invariant complex cells. The responses of simple cells are obtained similarly to Eq (3), but now with the previous DEM luminance-only channel (*l*) complemented by three new luminance/colour channels: *c* ∈ {*l*, *r*, *g*, *b*} with *r* = *R*+*G*/4+*B*/4, *g* = *R*/4+*G*+*B*/4, *b* = *R*/4+*G*/4+*B* and *l* as in L-DEM (see §3). This represents luminance-colour sensitive cells with different RGB component predominance, with the *l* channel representing luminance-predominant cells using physiologically perceived colour weights (corresponding to the Y channel of the XYZ colour space). Responses of the left and right RFs of binocular simple cells ($v_{L}^{\mu ,c}$ and $v_{R}^{\mu ,c}$) are obtained by convolving (*) the RFs with the corresponding left and right images $I_{L,R}^{c}(x,y)$:

$$\begin{array}{c} v_{L,R}^{\mu ,c}\left(x,y\right)=I_{L,R}^{c}\left(x,y\right)*\rho_{L,R}^{\mu ,c}\left(x,y\right). \end{array}$$ (11)

The augmented parameter set results in an encoding population of 8_*θ*_ × 3_*σ*, *f*_ × 2_*ϕ*_ × 60_Δ*x*_ × 1_Δ*ϕ*_ × 3_*μ*_ × 4_*c*_ = 34, 560 binocular simple cells (17,280 complex cells), twelve times larger than L-DEM due to the three different viewpoints *μ* and four luminance/colour channels *c*.

### 4.2 Disparity decoding population

The implementation uses the same decoding method as L-DEM, as specified in §3.2. However we are processing each of the four colour channels *c* independently—this allows us to show the benefits of colour without having to train the population again.

For each (*x*, *y*), the correlation (Corr) coefficient is now calculated between $\Psi_{\mu ,c}^{\text{sp}}$ and $W_{f,\theta ,\Delta x}^{\Delta x_{\text{stim}}}$. The correlation vector *r*_Δ*x*_stim_, *μ*, *c*_ now holds 60 × 3 × 4 cell responses, 60 for each *μ* and *c* combination. At this step, three viewpoint–based $D_{\mu }^{\text{LC}}$ disparity maps are built independently (examples are shown in Fig 2f-2h). The disparities assigned to each position (*x*, *y*) will be the values *d*_*μ*_ of the maximum correlations, where $d_{\mu }=\underset{\Delta x_{\text{stim}}}{\text{arg max}}{\left(r_{\Delta x_{\text{stim}},c}\right)}_{\mu }$, over all Δ*x*_stim_ and *c* values, for each *μ*. This yields three different disparity maps $D_{\mu }^{\text{LC}}(x,y)=d_{\mu }$. Biologically this corresponds to an activation of a single disparity cell per pixel and per viewpoint *μ*.

#### Viewpoint correction layer

Outputs from cell layers $D_{\mu }^{\text{LC}}$ are combined in a viewpoint correction layer, where the information from the three viewpoint disparity maps is used to select the most accurate information. This can be seen as a fusion of the disparity maps relative to the perspective of an observer with a left-side viewpoint. It is done by shifting the maps to the right accordingly (each pixel’s shift distance depends on its disparity value) and by computing the median M[·, ·, ·] of the three maps:

$$\begin{array}{ccc} {\hat{D}}^{\text{LCV}}\left(x,y\right) & = & \text{M}[D_{\text{Left}}^{\text{LC}}\left(x,y\right),\\ & & D_{\text{Centre}}^{\text{LC}}(x+0.5\;D_{\text{Centre}}^{\text{LC}}\left(x,y\right),y),\\ & & D_{\text{Right}}^{\text{LC}}(x+D_{\text{Right}}^{\text{LC}}\left(x,y\right),y)]. \end{array}$$ (12)

**Fig 3:**
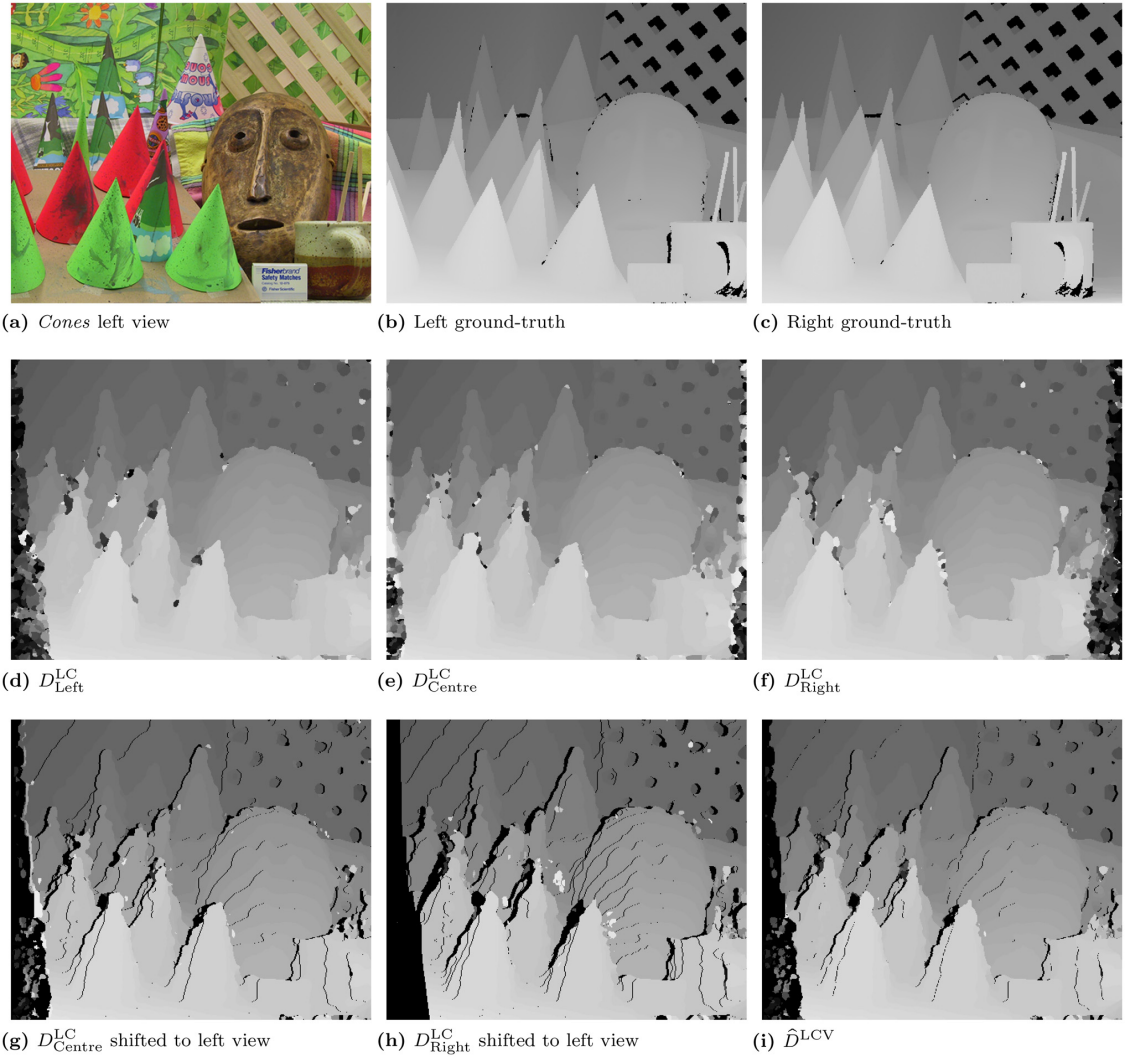
Example of viewpoint correction results for the *cones* [27] stereo pair. **(a)**: *Cones* left view of the pair. **(b)**: Left viewpoint ground-truth. **(c)**: Right viewpoint ground-truth. **(d)**: Left–viewpoint $D_{\text{Left}}^{\text{LC}}$. **(e)**: Centre–viewpoint $D_{\text{Centre}}^{\text{LC}}$. **(f)**: Right–viewpoint $D_{\text{Right}}^{\text{LC}}$. **(g)**: Centre to Left viewpoint disparity shift. **(h)**: Right to Left viewpoint disparity shift. **(i)**: Fusion of the three Left (shifted) maps into ${\hat{D}}^{\text{LCV}}$. Images (a), (b) and (c) are reprinted from Scharstein and Szeliski [26] under a CC BY license, with permission from Daniel Scharstein, original copyright 2003.

The resulting map can be seen in Fig 2(i). Combining viewpoints effectively increases the accuracy of disparity estimates at the left and right borders of objects, which are usually inaccurate due to viewpoint occlusion (i. e., each eye will see some information that the other does not). This leads to a correspondence problem, which is greater when the distance between left and right images of the pair is larger. For illustration purposes, Fig 3 shows a better example of the benefits of combining viewpoints, for the *cones* stereo pair. Here, the left and right images are more separate, with a maximum disparity of 59 pixels vs. only 15 pixels for *tsukuba*. *Cones*’ disparity maps highlight the greater differences between viewpoints. The fusion of all three maps is shown in image (i), where black pixels represent uncertain disparity regions, which we address below.

#### Background and occlusion correction layer

The map ${\hat{D}}^{\text{LCV}}(x,y)$ needs to be corrected in order to eliminate uncertain/unknown disparities due to incorrect disparity assignments in background regions or from occluded regions where disparities were shifted. To remove these, we use a two-step approach: First, we determine which disparity is the probable background and assign it as the farthest disparity in ${\hat{D}}^{\text{LCV}}(x,y)$. Computationally, this process is done in four steps:

(a) Count how many cells (*N*_*d*_) are activated per disparity value *d* ∈ {0, …, 59};
(b) normalise the counts by dividing each value by the square of the respective disparity: ${\hat{N}}_{d}=N_{d}/d^{2}$ (this gives less priority to the nearest/highest disparities, since it is expected that the background should be farthest);
(c) the background disparity is chosen as $d_{\text{bck}}=\underset{d}{\text{arg max}}\left({\hat{N}}_{d}\right)$; and
(d) to every disparity value ${\hat{D}}^{\text{LCV}}(x,y)<d_{\text{bck}}$ is assigned the value *d*_bck_.

Afterwards, remaining inactive disparity cells receive the minimum median cell value of the closest active disparity cells in the epipolar plane, yielding *D* ^LCV^(*x*, *y*). Results for *tsukuba* are shown in Fig 2(j). The result is much better when compared to Fig 2(i).

### 4.3 Experimental results

We tested the LCV-DEM on the same Middlebury stereograms used in L-DEM [26–28]. Fig 2(f) to 2(h) illustrate disparity maps for *tsukuba*—the $D_{\text{Left}}^{\text{LC}}$, $D_{\text{Centre}}^{\text{LC}}$ and $D_{\text{Right}}^{\text{LC}}$ images show results after luminance/colour grouping with three different viewpoints: Left, Centre and Right. The ${\hat{D}}^{\text{LCV}}$ shows the integration of the three viewpoint maps into a single Left viewpoint (Fig 2i), and *D* ^LCV^ shows the final LCV-DEM map after the background and occlusion correction layer (Fig 2j).

The quantitative results from the Middlebury stereo evaluation are discussed in §6, comparing L-DEM with LCV-DEM. We can visually verify (see Fig 2c and 2j) that there are several improvements from *D* ^L^ to *D* ^LCV^, nevertheless, the edges and regions around objects still lack a precise boundary definition. In the next section we will explain a complementary stereo model to assign disparity to line and edge features, and show how the integration of both disparity maps can be achieved.

## 5. Boundary enhanced LCVB-DEM

Another role for monocular simple and complex cells in V1 is the ability to extract multiscale lines and edges that are significant for object categorisation and recognition [19]. If lines and edges are extracted in V1, where left and right retinal projections are close together, one might even assume that depth is attributed to them. In other words, a “3D wire-frame representation” could be built in V1 for handling 3D objects and scenes. Although this idea is speculative, many V1 cells have been found to be tuned to different combinations of frequency (scale), orientation, colour and disparity. If not coded explicitly, disparity could be coded implicitly. This allows us to develop an alternative disparity model, where we assume that lines, edges and disparity are coded explicitly—the Line and Edge Disparity Model (LEDM).

Since disparity along object borders is the biggest problem for the presented DEM models, we also integrate at this step a low-level object salience model [17] that complements line and edge information from LEDM. This allows us to combine edge conspicuity with line/edge disparity information readily available in V1/V2. Using both on top of the LCV-DEM allows us to correct disparity values astride object borders. This yields our final model, the Luminance, Colour, Viewpoint and Boundary enhanced Disparity Energy Model (LCVB-DEM).

### 5.1 Line and Edge Disparity Model

**Fig 4:**
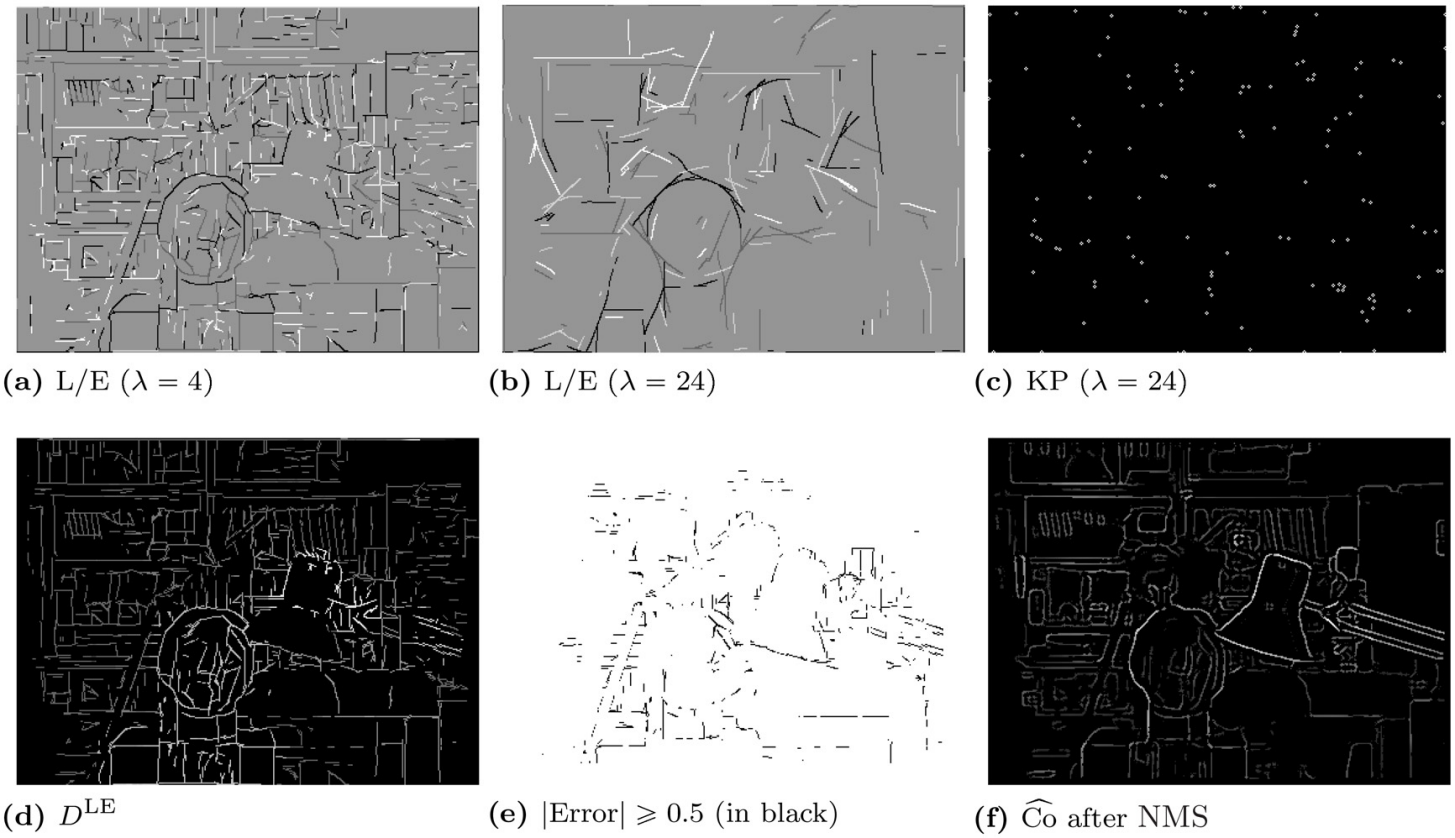
Line and edge disparity, and conspicuity results. **(a, b)**: Multiscale line and edge coding at *λ* = 4 and *λ* = 24. **(c)**: Keypoint map at *λ* = 24. **(d)**: *D* ^LE^ map with brightness-coded disparities. **(e)**: Bad pixels (black) with an absolute disparity error ≥ 0.5 [8]. **(f)**: Conspicuity map $\hat{\text{Co}}$ after applying lNMS.

Line and edge detection is based on responses of even and odd monocular simple cells, corresponding to the real and imaginary parts of a Gabor filter [19]. These responses are denoted by $R_{E}^{s,i}(x,y)$ and $R_{O}^{s,i}(x,y)$, with scale *s* given by *λ* and orientation *i* according to *θ*. We used the same 8 orientations as for the binocular cells in the previous models, and scales *s* corresponding to 4 ≤ *λ* ≤ 24 with a step size Δ*λ* = 2. Positive/negative *lines* are detected where *R*_*E*_ has a local maximum/minimum and *R*_*O*_ has a zero crossing. For *edges*, the even and odd responses are swapped. In total, there are four possibilities for positive and negative Line/Edge features (L/E). An improved scheme [19] consists of combining responses of monocular simple and complex cells, i. e., simple cells serve to detect positions and L/E types, whereas complex cells are used to increase confidence. Monocular complex cell responses are modelled by the modulus $C^{s,i}\left(x,y\right)={\left[{\left\{R_{E}^{s,i}\left(x,y\right)\right\}}^{2}+{\left\{R_{O}^{s,i}\left(x,y\right)\right\}}^{2}\right]}^{\frac{1}{2}}$. Spurious cell responses beyond line and edge terminations are suppressed by lateral and cross-orientation inhibition, and assemblies of grouping cells serve to improve L/E continuity in the case of curved L/Es. We denote the line and edge cell map by LE^*s*^(*x*, *y*). Fig 4 shows in (a) and (b) the multiscale line and edge coding for the *tsukuba* stereogram, at fine (*λ* = 4) and coarse (*λ* = 24) scales. Different grey levels, from white to black, show detected L/Es: positive/negative lines and positive/negative edges, respectively. We can see that many small L/Es are detected at fine scales, whereas coarse scales highlight global structures.

Keypoint maps are also exploited in the LEDM model, as these code line and edge crossings, singularities and points with large curvature. They are built from two types of end-stopped cells, single and double, which are modelled by the first and second derivatives of *C* ^*s*, *i*^. End-stopped responses are refined by tangential and radial inhibition to obtain precise keypoint cell maps KP^*s*^(*x*, *y*) [35]. Fig 4(c) shows the *tsukuba* keypoint map at a coarse scale (*λ* = 24).

The disparity assigned to each L/E is based on a left–right correspondence over scales:

1. First, we suppress L/Es which may be due to noise: at each scale *s* of the left and right maps ${\text{LE}}_{L,R}^{s}(x,y)$, we compute the maximum response of the monocular complex cells *C* ^*s*, *i*^ where L/Es have been detected. Any L/Es with a small amplitude (*C* ^*s*, *i*^ below 5% of the maximum response) are inhibited, yielding ${\hat{\text{LE}}}_{L,R}^{s}(x,y)$. The 5% threshold is necessary to eliminate detected L/Es at small gradients that do not represent region transitions. This value depends on the noise sensitivity of the Gabor responses and it was empirically determined. We found 5% to be consistently stable across many cases.
2. In the left map, at each L/E position (*x*_*L*_, *y*_*L*_) and at the finest scale (*s* = 1), ${\hat{\text{LE}}}_{L}^{1}$ is used to define regions of interest which are centred on each L/E position (*x*_*L*_, *y*_*L*_). These regions are formed by grouping cells with circular RFs. At the *same* position (*x*_*L*_, *y*_*L*_), other grouping cells are activated at all other scales, still in the left cell map, with RF sizes depending on the scale: 2 *λ*_*s*_. This *scale space* of the left cell maps (or hierarchical set of grouping cells with RFs at all L/E positions from *s* = 1) is used to accumulate displacement evidence of similar L/Es at similar scales, but with relative (shifted) positions in the right cell maps, ${\hat{\text{LE}}}_{R}^{s}$; see below.

Basically, the RFs serve to compare L/Es in the left and right cell maps as a function of the shift along the epipolar plane. This is done at all individual scales, after which the scales are combined. The right scale space ${\hat{\text{LE}}}_{R}^{s}$ can shift Δ*x* (epipolar line) with a step size *δx* = 1, such that 0 ≤ Δ*x* ≤ 59, for a total of 60 shifts, at which the L/Es in both scale spaces are binocularly compared, according to specific rules: the Δ*x* with the maximum L/E *correspondence* (defined below) is then assigned to the disparity map *D* ^LE^(*x*, *y*), where (*x*, *y*) still corresponds to L/E positions (*x*_*L*_, *y*_*L*_) of ${\hat{\text{LE}}}_{L}^{1}$ (Left viewpoint).

Computationally, at each scale and within each RF, four *correspondence* measures are combined with different weight factors:

(*M*_1_) Counting all line/edge L/Es with the same position, the same type (L or E) and the same polarity (+ or –);

(*M*_2_) As in *M*_1_ but only counting matching L/Es irrespective of type and polarity;

(*M*_3_) Counting the number of complex cells with similar amplitudes at all L/E positions, i. e., $|C_{L}^{s,i}-C_{R}^{s,i}|\leq 2$;

(*M*_4_) Counting the number of keypoints with about the same coordinates in ${\text{KP}}_{L,R}^{s}$, i. e., in small cell clusters of size 3 × 3.

Before combining the four measures, they are first normalised: *M*_1_, *M*_2_ and *M*_3_ are divided by the number of L/Es in ${\hat{\text{LE}}}_{L}^{s}$, whereas *M*_4_ is divided by the number of keypoints in ${\text{KP}}_{L}^{s}$, each number being computed within each respective RF. The normalised numbers are denoted by ${\bar{M}}_{i}$ and the final *correspondence* is determined by combining the weighted and normalised measures over all scales:

$$\begin{array}{c} {\hat{C}}_{\Delta x}=\sum_{s}\left(4\;{\bar{M}}_{1}+{\bar{M}}_{2}+{\bar{M}}_{3}+4\;{\bar{M}}_{4}\right). \end{array}$$ (13)

The weights for each factor were empirically determined after several trials. Finally, the horizontal disparity Δ*x* belonging to the maximum $\hat{C}$ value is stored in the depth map *D* ^LE^(*x*, *y*). For more implementation details see Rodrigues et al. [10].

LEDM was applied to the Middlebury stereo pairs, exemplified with *tsukuba* in Fig 4. The results were very good, with disparities correctly assigned to object borders in image (d). The disparity error image (e) displays the incorrect values as black pixels, showing that almost all lines and edges have a correctly assigned disparity (80.7% at a 0.5 max error and 90.6% at a 1.0 max error).

### 5.2 Line and Edge region enhancement

To enhance disparity accuracy in line and edge regions and to remove small gaps we combine LCV-DEM with LEDM into an intermediate representation ${\hat{D}}^{\text{LCVE}}$, similar to Rodrigues et al. [10]. For each L/E pixel in the *D* ^LE^ map we define a small cluster at the L/E position plus its N_4_ neighbourhood (left, right, top and bottom neighbours) and compare its median to the median of a similar cluster in *D* ^LCV^, at the same position. If the clusters have similar median values (less than a threshold *t*), the *D* ^LCV^ cluster response at the L/E position is propagated into ${\hat{D}}^{\text{LCVE}}$ as detailed below. Mathematically, ∀(*x*, *y*),

$$\begin{array}{c} |\text{med}({\text{N}}_{4}[D^{\text{LE}}\left(x,y\right)])-\text{med}({\text{N}}_{4}[D^{\text{LCV}}\left(x,y\right)])|\leq t\;, \end{array}$$ (14)

where *t* ∈ {1,…,5} is an integer value that represents the maximum allowed difference and med(·) the median. If Eq (14) is *false*, then the *D* ^LCV^ cluster response is assumed to be wrong, and its region is filled in ${\hat{D}}^{\text{LCVE}}$ using the value of *D* ^LE^(*x*, *y*). This way, we correct the LCV-DEM results using the LEDM responses. This process starts with *t* = 1 and it is applied in several cell layers, recursively, on top of the newly created ${\hat{D}}^{\text{LCVE}}$ map, i. e., if it is not possible to fill it any more, but there are still gaps, we increment *t* by 1 and repeat the same procedure. In our experiments 5 was the maximum value. Biologically, this could correspond to 5 layers of ${\hat{D}}^{\text{LCVE}}$ that activate neighbouring “idle” cells. The result can be seen in Fig 2(k), where many small regions have been corrected.

### 5.3 Object Boundary enhancement

Despite the above process to correct ambiguous regions, some boundaries can still be improved. In real scenes, disparity borders are mostly found at the contours of real objects, so we use a disparity sharpening process based on local contrast of disparity values, conspicuity information and line/edge boundaries to reach the final stage of this whole process, yielding *D* ^LCVB^—Luminance, Colour, Viewpoint and Boundary enhanced Disparity Energy Model (LCVB-DEM). This process requires three steps:

#### Edge conspicuity

In general, object borders are perceptually salient in a scene. In order to detect them, we first define *edge conspicuity* $\tilde{\text{Co}}(x,y)$ as a low-level V1 process. Mathematically, it is the maximum difference between colours in $I_{L}^{c}(x,y)$, with *c* ∈ {*l*, *r*, *g*, *b*}, at four pairs of symmetric positions with pixel distance $\|{\vec{\delta }}_{i}\|=1$ from point (*x*, *y*), i. e., on horizontal, vertical and two diagonal lines [17]. Conspicuity $\tilde{\text{Co}}(\xi )$ is the maximum Euclidean distance of all four colour pairs,

$$\begin{array}{c} \tilde{\text{Co}}\left(\xi \right)=\max_{i=1}^{4}\sqrt{\sum_{c}{\left[I_{L}^{c}(\left(x,y\right)-{\vec{\delta }}_{i})-I_{L}^{c}(\left(x,y\right)+{\vec{\delta }}_{i})\right]}^{2}}. \end{array}$$ (15)

In order to remove low responses due to small colour gradients that do not represent edges, responses lower than 10% of $\text{max}(\tilde{\text{Co}})$ are inhibited. A value of 10% for this threshold was found to be a consistently good choice for many cases. This value is linked to the perceptual nature of differentiating colours and is an empirically determined constant. We can think of this inhibition process as following the Weber-Fechner law (just-noticeable differences) in psychophysics, with this threshold being Weber’s constant. The remaining active cells are selected by Non-Maximum Suppression (NMS), which yields conspicuity edge positions $\hat{\text{Co}}$. Fig 4(f) shows the *tsukuba* $\hat{\text{Co}}$ map after NMS.

#### Border Detection

We use a specific binary border-detection cell layer *B*_*d*_ that combines cell responses from $\hat{\text{Co}}$, *D* ^LE^ and *D* ^LCV^. *B*_*d*_(*x*, *y*) cells are only active when the following condition is *true*: $\forall (x,y):\hat{\text{Co}}(x,y)>0\vee [D^{\text{LE}}(x,y)>0\wedge |D^{\text{LE}}(x,y)-D^{\text{LCV}}(x,y)|>0]$, i. e., at conspicuous borders and at lines/edges when they correspond to object borders and not to homogeneous disparity regions. Then, we devise two approaches to detect and correct bad disparity estimations by analysing regions that are *far* or *near* *B*_*d*_ active cells:

- The *far* case will cover regions where there are no active *B*_*d*_ cells nearby, i. e., regions that should have a homogeneous disparity value. Here we analyse relationships between small disparity *peaks* or *bumps* and their surrounding areas. For *peaks*, if the inside median disparity of a small cell cluster (10px radius) *M*_in_ is different from that of its border (outside perimeter) *M*_out_, and if *M*_in_ > *M*_out_ then the cell cluster disparity is reduced to the value *M*_out_, eliminating the disparity peak. For *bumps*, if *M*_in_ < *m*_out_, with *m*_out_ the minimum value of the border region (perimeter), then the cell cluster disparity is increased to *m*_out_, slightly bumping the disparity depression to a coherent region background value (using *M*_out_ here could lead to wrong results near regions with objects, as bumps could wrongly be raised to their disparity instead).
- For regions *near* active *B*_*d*_ cells, i. e., near object borders, every active border in *B*_*d*_ activates a filling in process. We assume that the entire disparity map *D* ^LCV^ is covered by overlapping Φ cells with RFs of 3 × 3 pixels and one pixel distance between their centres, which compute the median disparity in their RF. On each side and orthogonal to a *B*_*d*_ edge, a cluster of three orthogonal neighbouring Φ cells starts close to the edge and moves until a maximum distance of 25 pixels. If the three neighbouring cells are denoted by Φ_1_ (closest to border), Φ_2_ (middle) and Φ_3_ (farthest from border), then disparity Φ_2_ is propagated to the border at the first position where |Φ_2_ − Φ_3_| ≥ 2 and Φ_2_ = Φ_1_. Hence, a stable disparity value (before the first significant disparity transition) is propagated until a *B*_*d*_ edge. In this process we apply median disparities in order to skip disparity changes which do not likely correspond to true object borders.

The completion of both approaches returns an enhanced disparity map *D* ^LCVE^; see Fig 2(l).

#### Median smoothing

Finally, the last step serves to correct all locally inconsistent disparities by assigning to each (*x*, *y*) position the most probable disparity within a small RF. This process is similar to a median smoothing filter and is achieved by applying circular cell clusters to *D* ^LCVE^(*x*, *y*) (6px radius; slightly bigger or smaller sizes do not affect the global ranking in the Middlebury test, despite slightly improving/degrading individual images). This yields the final disparity map LCVB-DEM denoted by *D* ^LCVB^, shown in Fig 2(m).

### 5.4 LCVB-DEM Experimental Results

Fig 2 shows the *D* ^LCVE^ map in (l) and the final disparity map *D* ^LCVB^ in (m). By subjecting the last result to the Middlebury evaluation test we obtain the *“Bad pixels absolute disparity error ≥ 0.5”* and *“Signed disparity error”* of *D* ^LCVB^, respectively shown in (n) and (o). When comparing (m) with the results obtained from L-DEM in (c) we can observe significant improvements. Nevertheless, the number of pixels with wrong disparity estimates, although reduced, is still significant (see Fig 2n, at regions near depth discontinuities) and the biggest errors are located at the border of the desk-lamp and its support (Fig 2o).

**Fig 5:**
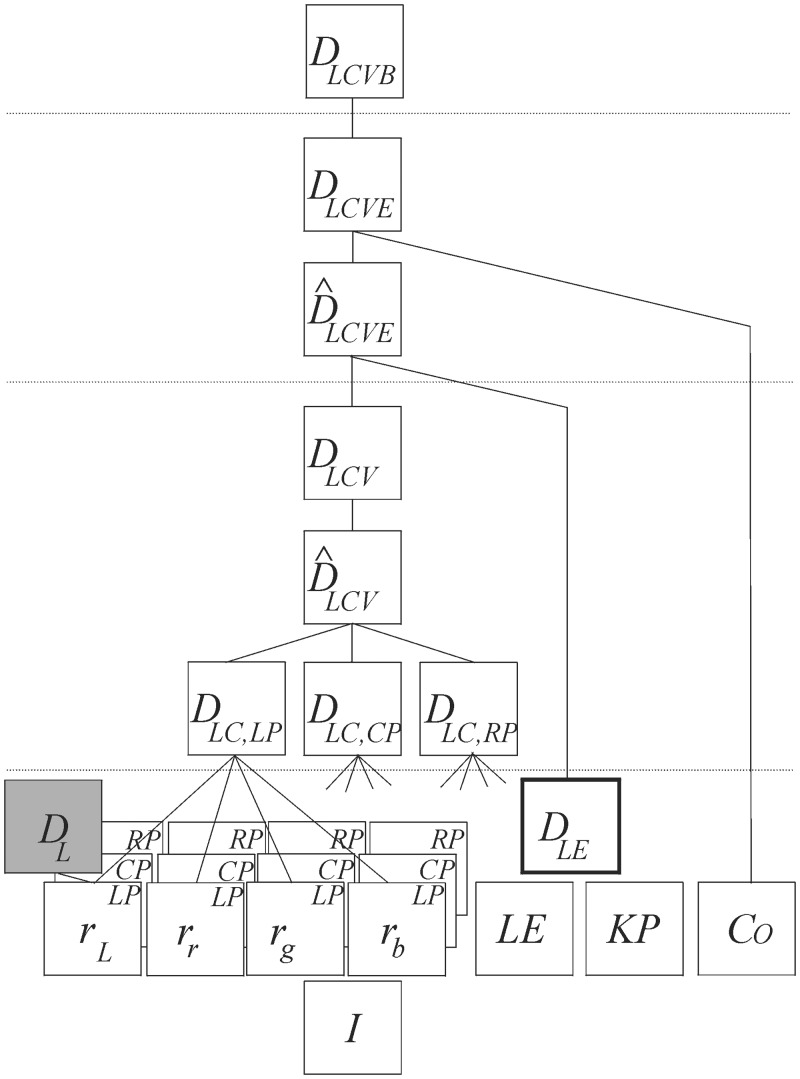
Summary of the different features and disparity maps leading to the LCVB-DEM.

Fig 5 details schematically all intermediate disparity maps needed to create the LCVB-DEM model, divided into three big layers. Our first DEM implementation from Martins et al. [7] (detailed in §3 as the L-DEM) is highlighted in grey. In the next section we will show results for other images and discuss the different disparity models qualitatively and quantitatively.

## 6. Results

**Fig 6:**
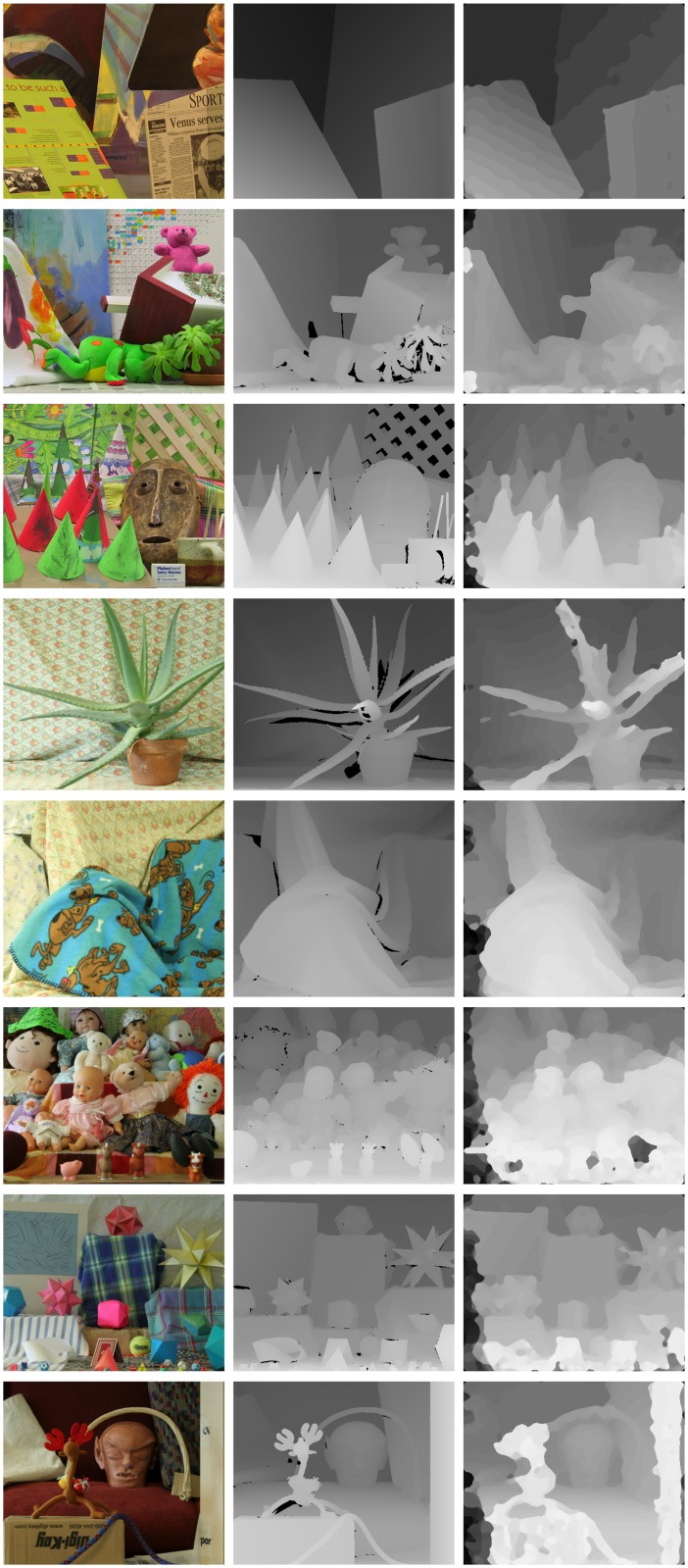
LCVB-DEM Middlebury dataset results. Each row shows the left view of each stereogram, its ground-truth, and final disparity result. Top-to-bottom:*venus*, *teddy*, *cones*, *aloe*, *cloth3*, *dolls*, *moebius* and *reindeer* stereograms. Images in the first and second columns are reprinted from Scharstein and Szeliski [26, 27], Scharstein and Pal [28] under a CC BY license, with permission from Daniel Scharstein, original copyright 2002–2006.

As mentioned in §3.3, we also tested the model at the different implementation steps on various stereograms, including *tsukuba*, *venus*, *teddy*, *cones*, *aloe*, *cloth3*, *dolls*, *moebius* and *reindeer* [26–28]. Fig 6 shows the left view of the pair along with the left groundtruth and our final LCVB-DEM result.

**Fig 7:**
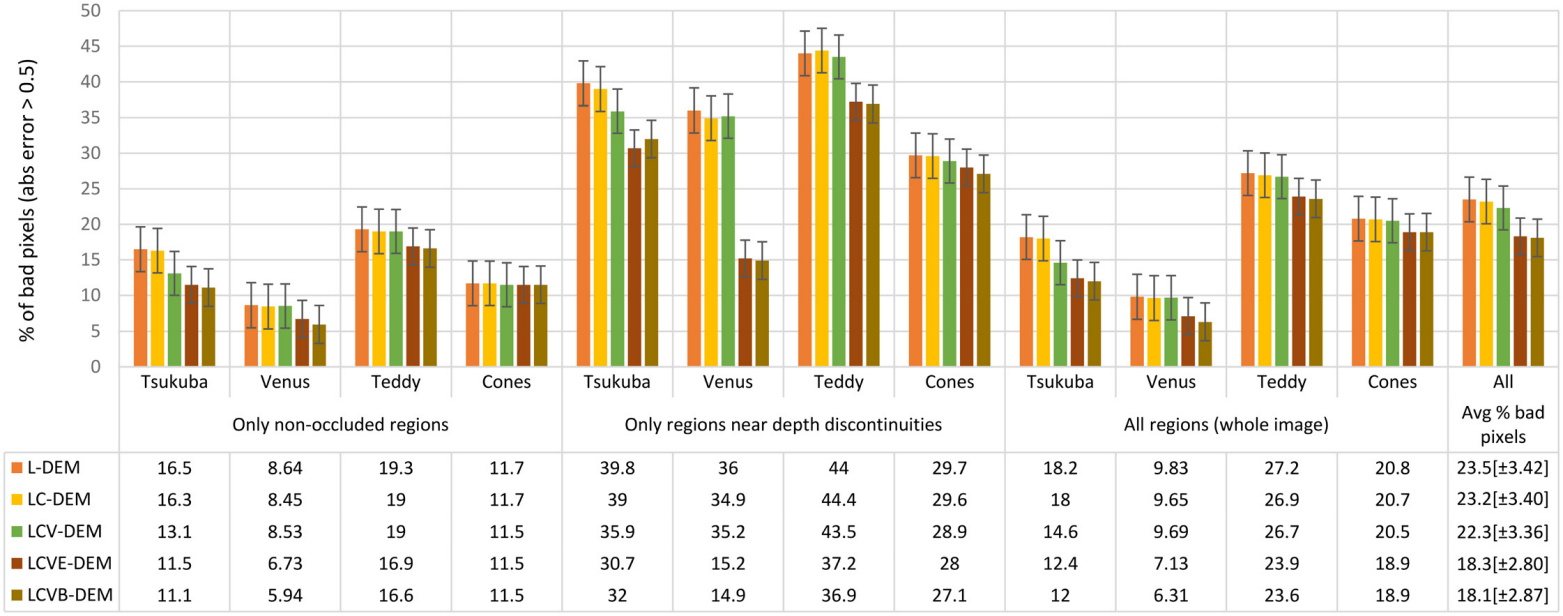
Evaluation of disparity errors, for the different model layers. Error bars represent the standard error of the mean for each model layer and are specified between brackets in the last column.

Fig 7 shows the evolution of error results, using the Middlebury evaluation page [8], for *tsukuba*, *venus*, *teddy* and *cones*, at different steps of the model which correspond to the final disparity maps in each layer (see Fig 5). In the bottom table of Fig 7, top to bottom are shown: (1) L-DEM; (2) Luminance and Colour Disparity Energy Model (LC-DEM) which includes colour but not viewpoint correction; (3) LCV-DEM with colour and viewpoint; (4) LCVE-DEM with colour, viewpoint and LEDM; and finally (5) LCVB-DEM which integrates all above steps with object border enhancement. The results show improvements in all layers of the model, with the number of error pixels mostly decreasing consistently.

We can see that our model performs best in non-occluded regions but it is not as good near depth discontinuities. This was expected, because L-DEM and LCV-DEM struggle at border transitions, which is why the LEDM model is used to improve the LCV-DEM; it improves results but without yet achieving outstanding results—still, the error for regions near depth discontinuities decreases more than a factor of two in the *venus* case. The *all regions* columns refer to entire images, even regions which are half-occluded. *Avg % bad pixels* gives a general indication of how well the methods perform, as it shows the average percentage of bad pixels (wrong estimates) over all twelve columns. In all cases, the bad pixels were counted by applying the smallest error criterion possible: a disparity difference with the ground-truth greater than 0.5; for details see Scharstein and Szeliski [8].

**Fig 8:**
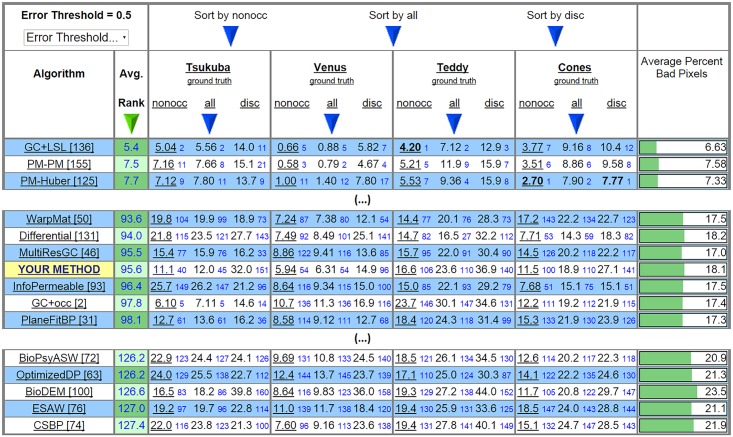
Middlebury Stereo Evaluation table [8], with methods ordered by total Average Rank using the strictest error threshold (0.5). LCVB-DEM is listed as “YOUR METHOD ” and the original L-DEM method is listed as “BioDEM”. The small blue numbers beside each column indicate the Average Rank of each individual result against all other methods. The table was retrieved on *13th January 2015*.

Overall, best results were obtained for images without many small details. This is related to the size of the RFs in the cell population; smaller RFs are required to resolve the smallest details, but unfortunately they also increase binocular correspondence errors. Fig 8 shows our result when compared to the ranked results of other methods, which can include more sophisticated post-processing and top-down methodologies, like image segmentation, for yielding massively improved pixel-to-pixel correspondences. This table was replicated from the Middlebury online evaluation webpage, applying the smallest available error threshold (≤ 0.5) to emphasise that a biologically-inspired algorithm can achieve competitive results.

We can also see that the LCVB-DEM method improves the results achieved with the L-DEM (*BioDEM*) method. Overall, we achieved a good position in the average ranking table: rank 95.6 between 5.4 (best) and 159.5 (worst), on a total of 162 evaluated methods. With LCVB-DEM we significantly rise 31 positions, from position 126.6 to 95.6 (table retrieved on *13th January 2015*) relative to *BioDEM*. If we average the rankings of individual results in the columns devoted to non-occluded regions, our method would rise 20.6 positions, to rank 75. This confirms that the biggest improvement can be achieved by even more accurate estimates near depth discontinuities. Finally, to the best of our knowledge, our method is ranked highest when compared with other biologically inspired methods [12–36].

## 7. Discussion

We presented a hierarchical model of four disparity estimation methods, based on the biological lDEM. It can achieve good results if compared with computer vision methods [8] and it advances the state-of-the-art of biologically inspired methods [12, 36]. The advantage of the proposed DEM approach is that it does not rely on extrinsic knowledge of cell parameters to estimate disparities, requiring only trained cell populations. All used DEM-like models rely on two neuronal populations: (1) an *encoding* population that learns to discriminate disparities from repeated presentations of random and binocularly uniform stimuli, resulting in a population activity code (i. e., a mean spike count) for each stimulus disparity; and (2) a *decoding* population that associates each code to a specific disparity value, using synaptic weights that store the mean activity of the population [1]. After foveal training, the populations are ready to evaluate disparities at any retinotopic (image) position, each local activity code being decoded into a single disparity value. Although not explored here, we also expect the decoding population to have some degree of neural plasticity and context-awareness, dynamically adapting itself to correlate the decoding weights to local image content.

All proposed models use a large number of cells: the L-DEM model starts with 2880 binocular simple cells which are combined into 1440 complex cells, at each retinotopic (image) position; LCVB-DEM increases that number to 17,280 complex cells. Nevertheless, these are trivial numbers when compared to total V1 size, estimated at about 190 million cells [37], but that number could well be near 243 million (average volume of V1 of 5,405 mm^3^ × 45,000 cells/mm^3^).

The role of colour in biological disparity models is still rather speculative [32], with little research into biological disparity models that employ colour, even in view of already existing evidence that disparity-sensitive neurons can also be isoluminant-sensitive [33, 34]. Meanwhile, our empirical evidence suggests that mixing colour weights may definitively play a significant role in improving the *luminance* discrimination of cells, which can significantly improve disparity estimations. Empirically, using different Y-channel luminance formulas in the XYZ colour space significantly affected the accuracy of the disparity maps, suggesting that the brain’s luminance pathway (where L- and M-cone responses are combined) plays a key role in the stereo matching process by maximising the differences between regions of a scene. This is expected evolutionarily, since the brain needed to develop a robust disparity system that worked well for various survival-related tasks, especially in the dark, when scotopic colour perception is unreliable. Nevertheless, colour can still play an important role in defining disparity transitions by highlighting conspicuous object borders [17].

The role of *perspective correction*, to shift the viewpoint of disparity maps in order to yield better estimates, is also biologically plausible: even uV1 cells display the ability to shift their RFs [38]. Basically, this process increases the robustness of binocular correspondence (i. e., stereo-matching) by combining the responses of three binocular RF perspectives, instead of just one, at each image position. This is especially useful for scenes with many occlusions or periodic textures. The method chosen for perspective shifting, shown in Eq (12), could also be particularly useful for combining many different perspectives in multi-view stereo. In this paper we considered the left view, but this was because of a practical reason. In biological vision models this should be the central view in order to mimic cyclopean vision and minimise object border occlusions between left/right perspectives.

A big advantage of the models is that they exploit cell types that are already available in the cortex: monocular simple cells can be paired to construct binocular cells. They are also useful for coding lines and edges, as in the lLEDM, or even for object segregation or brightness perception [19]. Also, as shown by Pugeault et al. [9], different spatial structures can be linked both in 2D and 3D by using constraints like good continuity. These structures can be complemented with other features, like optical flow, colour and texture, to help in object recognition. The LEDM exploits the structural organisation of V1 hypercolumns, with very close left and right retinal projections, associating depth to detected lines and edges at a low level, i. e., a sort of “wireframe” representation [1]. This is useful for post-processing of DEM estimates in occluded regions, where some detail is visible in one projection but not in the other. This allows the LCVB-DEM to use LEDM and conspicuity edges to steer and correct disparity estimations on both edge sides, while smoothing disparities in regions without edges. The role of phase tuning in sharpening edge disparities is also yet to be explored [11].

Finally, we propose and illustrate that the classical DEM (L-DEM) and LEDM can be used to create a disparity “gist” map, i. e., they are robust enough to quickly draft the environment, either from binocular energy complex cells or from object contours (the bottom layer of Fig 5). Such maps are sufficient for person or robot navigation, as they are based on quickly extracted visual features in a very low-level layer. In a second layer, the DEM is combined with colour and perspective correction, giving a more accurate disparity map, but still lacking well-defined borders around objects. In the third layer, information about edges is integrated into the LCVE-DEM disparity map. The fourth and final layer sharpens object borders using saliency data on top of LCVE-DEM, yielding LCVB-DEM. In summary, we have two disparity gist-like maps, one with localised edge information (LEDM) and one with spatially inaccurate, but precise region information (L-DEM), which are later combined with colour and viewpoint to form a more robust map (LCVB-DEM).

For further research, it makes sense to explore some alternative and promising combinations of binocular cells that proved to yield more biologically accurate disparity tuning curves in rhesus monkeys [4, 5]. The role of phase-tuned cells is also an interesting topic [19, 23], as their use can be seamlessly integrated into our model, signalling false disparity matches that can be immediately corrected at a low-level.

## Supporting Information

S1 Fig Tsukuba LCVB-DEM result. (PNG)

S2 Fig Venus LCVB-DEM result. (PNG)

S3 Fig Teddy LCVB-DEM result. (PNG)

S4 Fig Cones LCVB-DEM result. (PNG)

S5 Fig Aloe LCVB-DEM result. (PNG)

S6 Fig Cloth3 LCVB-DEM result. (PNG)

S7 Fig Dolls LCVB-DEM result. (PNG)

S8 Fig Moebius LCVB-DEM result. (PNG)

S9 Fig Reindeer LCVB-DEM result. (PNG)
