# Supplementary figures and images for: Luminance, Colour, Viewpoint and Border Enhanced Disparity Energy Model
Source: PLoS One. 2015 Jun 24;10(6):e0129908. doi: 10.1371/journal.pone.0129908 (PMC4480855; doi:10.1371/journal.pone.0129908)

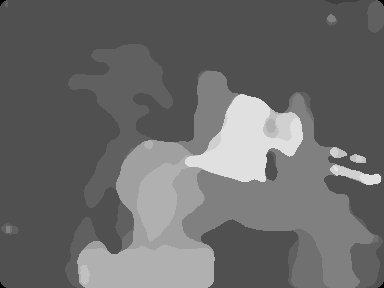

Supplement: S1 Fig — (PNG) [file pone.0129908.s001.png]

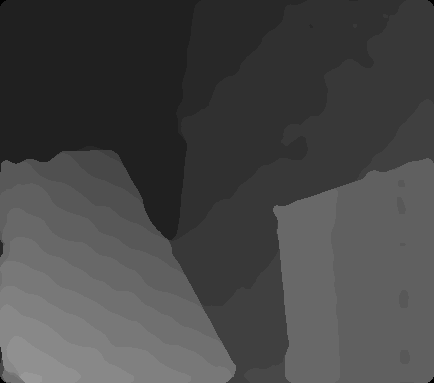

Supplement: S2 Fig — (PNG) [file pone.0129908.s002.png]

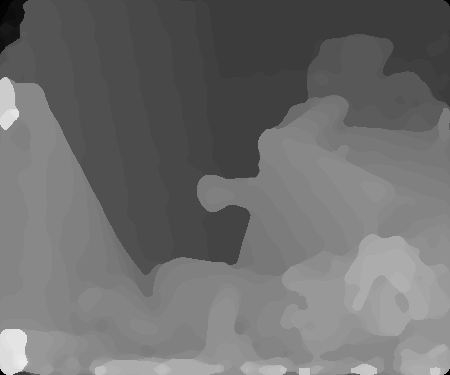

Supplement: S3 Fig — (PNG) [file pone.0129908.s003.png]

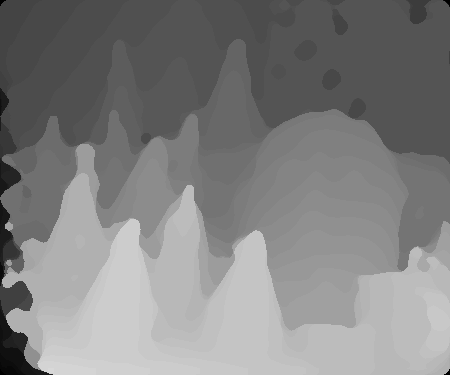

Supplement: S4 Fig — (PNG) [file pone.0129908.s004.png]

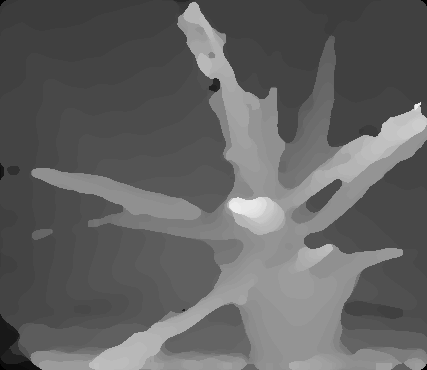

Supplement: S5 Fig — (PNG) [file pone.0129908.s005.png]

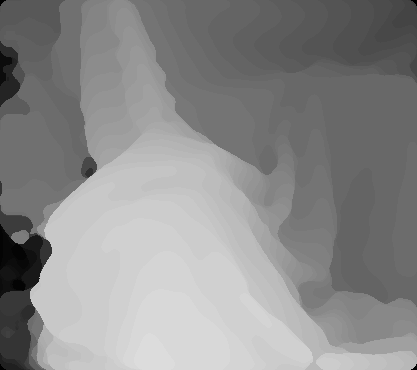

Supplement: S6 Fig — (PNG) [file pone.0129908.s006.png]

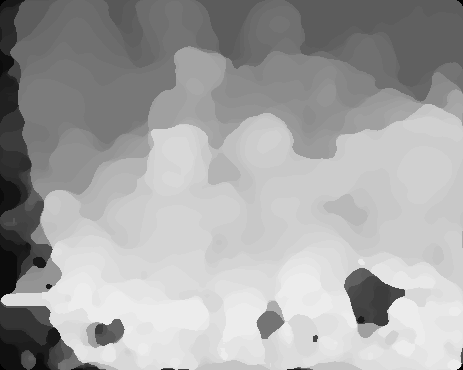

Supplement: S7 Fig — (PNG) [file pone.0129908.s007.png]

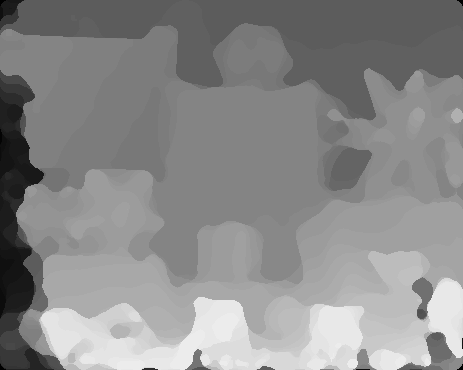

Supplement: S8 Fig — (PNG) [file pone.0129908.s008.png]

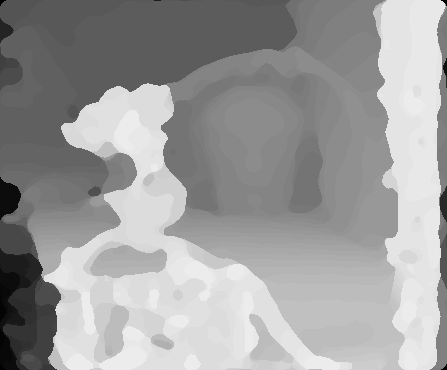

Supplement: S9 Fig — (PNG) [file pone.0129908.s009.png]
